# Effect of Particulate Matter in Atopic Dermatitis through HDACs and Filaggrin Alteration

**DOI:** 10.4014/jmb.2502.02047

**Published:** 2025-07-18

**Authors:** Yoon Jin Roh, Kui Young Park, Na Yeon Koo, Yong Hee Choi, Hye Won Song, Sin Woo Sung, Ka Ram Kim, Seong Jun Seo, Joon Seok, Mi-Kyung Lee

**Affiliations:** 1 Department of Dermatology, Chung-Ang University Hospital, Seoul 06973, Republic of Korea; 2 Department of Laboratory Medicine, Chung-Ang University Hospital, Seoul 06973, Republic of Korea

**Keywords:** Atopic dermatitis, filaggrin, histone deacetylation, particle matter, trichostatin A

## Abstract

Atopic dermatitis (AD) is a skin condition that can be exacerbated by particulate matter (PM). The primary causes of AD are believed to be impairments in the skin barrier, such as filaggrin (FLG) abnormalities. Although there is substantial evidence for genetic factors contributing to AD, it is challenging to attribute the disease's predisposition solely to genetics. We hypothesize that PM may induce epigenetic modifications in AD, impacting FLG expression. Here, we assessed histone deacetylases (HDACs) and FLG levels under AD conditions with or without exposure to PM using qRT-PCR, western blotting, and immunofluorescence. Additionally, we observed changes in these molecules when co-treated with the HDAC inhibitor, trichostatin A (TSA). FLG levels tended to decrease when PM or IL-4/13 were given individually, and a further decrease upon IL-4/13 and PM cotreatment. Interestingly, HDAC3 and HDAC6 are increased when they were both given PM10 and IL-4/13 co-treatment compared to IL-4/13 treatment alone. FLG levels exhibited a significant restoration and the levels of HDACs were changed when treated with TSA than with IL-4/13 or IL-4/13+PM co-treatment. We found changes in FLG and HDACs in the AD-like in vivo model and in this model with PM. Our findings suggest that PM can induce epigenetic alterations in AD. Treatment with TSA ameliorates these effects on FLG expression, indicating its potential as a novel therapeutic approach for AD.

## Introduction

Atopic dermatitis (AD) is a chronic, relapsing inflammatory skin disorder characterized by intense pruritus, eczematous lesions, and impaired epidermal barrier function [1]. A key contributor to AD pathogenesis is the loss or dysfunction of filaggrin (FLG), a structural protein essential for skin hydration, pH regulation, and protection against external irritants [2]. Mutations in the *FLG* gene are frequently identified in patients with AD across various ethnic groups, including East Asians [3]. Reduced FLG expression compromises the skin barrier, increases transepidermal water loss, and facilitates allergen and irritant penetration, triggering immune activation and inflammation [4].

The *FLG* expression is positively regulated by transcription factors such as ovo-like zinc finger 1 (OVOL1), Kruppel-like factor 4 (KLF4), and peroxisome proliferator-activated receptors (PPARs), which promote keratinocyte differentiation and skin barrier formation [5]. Conversely, Th2 cytokines, including interleukin-4 (IL-4) and IL-13 are highly expressed in AD lesions and downregulate *FLG* via the IL-4Rα/Signal Transducer and Activator of Transcription 6 (STAT6) signaling pathway [6-8]. This inhibitory mechanism is substantiated by the oxazolone (OXA)-induced AD mouse model, where repeated topical exposure elicits Th2-dominant inflammation epidermal hyperplasia and *FLG* suppression [9, 10].

In recent years, particulate matter (PM) has emerged as an essential environmental aggravator of AD [11]. PM can penetrate or adhere to the skin surface inducing oxidative stress, inflammation and may disrupt gene expression through epigenetic mechanisms such as DNA methylation and histone modification [12, 13]. Given FLG’s sensitivity to environmental stimuli PM exposure may contribute to *FLG* suppression and further barrier breakdown [14].

Among epigenetic regulators, histone deacetylases (HDACs) are key mediators of transcriptional repression by removing acetyl groups from histone tails leading to chromatin condensation [15]. In the skin, HDAC3 regulates keratinocyte differentiation and immune responses [16-18], while HDAC6 participates in oxidative stress regulation and cytoskeletal remodeling [19-21]. A recent study by Jin *et al*. (2023) demonstrated that HDAC6 promotes skin inflammation via STAT3 acetylation [22], although its relationship with *FLG* expression or environmental triggers such as PM was not investigated [19, 23].

To the best of our knowledge, no studies have directly demonstrated how HDAC activation suppresses *FLG* expression and the mechanistic link to AD pathogenesis remains unclear. Experimental evidence on the roles of HDAC3 and HDAC6 in regulating *FLG* and contributing to AD pathophysiology is limited indicating the need for further mechanistic research.

While direct studies in AD are limited, HDAC overexpression and the therapeutic potential of HDAC inhibitors have been reported in other skin conditions, such as psoriasis and cutaneous T-cell lymphoma [24]. Pan-HDAC inhibitors such as trichostatin A (TSA) and suberoylanilide hydroxamic acid (SAHA) have effectively alleviated skin inflammation by restoring *FLG* expression and modulating immune function [18, 25-27]. However, the impact of TSA on PM-induced HDAC activation and *FLG* suppression remains unknown. Selective inhibitors such as tubastatin A (targeting HDAC6) and RGFP966 (targeting HDAC3) exhibit isoform-specific activity, supporting the therapeutic potential of both broad-spectrum and targeted HDAC modulation in AD [28].

This study aimed to determine whether PM exposure enhances HDAC3 and HDAC6 activity, leading to epigenetic suppression of *FLG* and exacerbation of AD pathology. Moreover, it was evaluated whether treatment with TSA can alleviate these effects, proposing a novel mechanism linking environmental and epigenetic factors in AD, and offering insights into HDAC-targeted therapies [3, 13-15, 21, 22, 25].

## Materials and Methods

### Reagents

Standard reference materials (SRM) 1649b (PM) were procured from the National Institute of Standards and Technology (NIST, USA) and suspended in serum-free medium. Trichostatin A (TSA) was acquired from Sigma-Aldrich (USA), dissolved in cell culture grade dimethyl sulfoxide (DMSO, PanReac AppliChem, GmbH), and subsequently diluted with DPBS (Welgene, Republic of Korea). Human epidermal keratinocytes, neonatal (HEKn) were sourced from Thermo Fisher Scientific (USA). IL-4 and IL-13, employed for generating the AD-induced cell model, were obtained from PeproTech (USA). For western blot analysis, anti-FLG antibody was purchased from LifeSpan Biosciences (USA), while histone deacetylase (HDAC) 3 and HDAC6 antibodies were acquired from Abcam (UK). Anti-GAPDH antibody was procured from Santa Cruz Biotechnology (USA). Anti-rabbit IgG, horseradish peroxidase (HRP)-linked antibody, anti-mouse IgG, and HRP-linked antibody were sourced from Cell Signaling Technology. 4-Ethoxymethylene-2-phenyl-2-oxazolin-5-one (oxazolone) was purchased from Sigma-Aldrich. Oxazolone was dissolved in the vehicle (acetone : olive oil = 4 : 1 mixture).

### Standard Reference Material (SRM) 1649b (Particle Matter; PM))

During the mid to late 1970s, NIST collected urban PM using a baghouse specially designed for the purpose from the Washington, DC, area. PM were collected over a period greater than 12 months and represents an atmospheric particulate matrix to validate analytical methods. SRM 1649b (urban dust) has been prepared from the bulk material (SRM 1649) collected at this time but it has been sieved to a smaller particle size with a mean diameter of 105 μm [29].

### Cell Culture

HEKn cells were maintained in EpiLife serum-free medium (Gibco, USA) supplemented with 1% Human Keratinocyte Growth Supplement (HKGS) (Gibco) at 37°C in a humidified incubator containing 5% CO_2_. Upon reaching confluence, cells were treated with TrypLE Select Enzyme (Gibco) for five min. Subsequently, cells were seeded at a density of 5 × 10^4^ cells/well in 6-well plates and cultured for 24 h. To inhibit HDAC in cells, TSA was dissolved in DMSO, diluted with DPBS, and administered at concentrations of 100, 200, and 300 μM for three h. PM treatment was carried out for 24 h.

### Generation of AD-Induced Cell Model

HEKn cells were seeded at a density of 3 × 10^4^ cells/well in 6-well plates and incubated for 24 h. IL-4 and IL-13 were dissolved in 10 μl of filtered deionized water (DW), followed by the addition of 990 μl of 0.1% BSA to achieve a concentration of 10 μg/ml. Cells were treated with a final concentration of 50 ng/ml by adding 10 μl/well.

### Quantitative Real-Time Polymerase Chain Reaction

Total RNA was extracted from HEKn cells utilizing TRIzol reagent (Invitrogen, USA) according to the manufacturer's instructions. cDNA synthesis for quantitative real-time PCR (qRT-PCR) was performed using the RevertAid First Strand cDNA synthesis kit (Thermo Fisher Scientific) with 1 μg of total RNA. qRT-PCR assays were conducted using a real-time thermal cycler (Applied Biosystems, USA) and PowerUp SYBR Green Master Mix (Applied Biosystems). All results were normalized to the housekeeping gene GAPDH. Relative quantification was executed using the comparative 2^-ΔΔCt^ method in accordance with the manufacturer's guidelines.

### Western Blot Analysis

HEKn cells were lysed with radioimmunoprecipitation assay (RIPA, Thermo Fisher Scientific) buffer supplemented with protease and phosphatase inhibitor cocktails (Invitrogen). For FLG-specific analysis, NP-40 cell lysis buffer (Invitrogen) containing PMSF and protease inhibitor cocktail was used instead. Following a 30 min incubation on ice, samples were centrifuged at 13,200 rpm for 20 min. Protein concentrations in the supernatants were determined using a bicinchoninic acid assay (BCA, Thermo Fisher Scientific), with absorbance measured at 562 nm on a microplate reader. Equal protein amounts were resolved on 8-10% SDS-PAGE gels and transferred to nitrocellulose (NC) membranes. The membranes were blocked in 5% skim milk dissolved in Tris-buffered saline (TBS, Biosesang, Republic of Korea) with added Tween-20 (TBS-T) for 1 h. Membranes were then incubated with primary antibodies overnight at 4°C, followed by HRP-conjugated secondary antibodies (1:5000 dilution) for 1 h at room temperature. Protein expression was visualized using the EzWestLumi plus system (ATTO, Japan), and images were captured with a ChemiDoc™ XRS image analyzer (Bio-Rad, USA).

### Immunofluorescence

HEKn cells were plated in 24-well plates containing serum-free medium supplemented with 1% HKGS. After 24 h, the medium was replaced with fresh serum-free medium, and the cells were incubated for an additional 3 h. Subsequently, the medium was removed and replaced with medium containing TSA. After treating the cells with TSA for 3 h to inhibit HDAC expression, they were washed with DPBS and treated with PM for 24 h. Cells were fixed with 4% paraformaldehyde (PFA, Biosesang), washed with DPBS, permeabilized using 0.5% Triton X-100 in PBS for five minutes, and blocked with 1% BSA. Primary antibodies for FLG (Santa Cruz), HDAC3 and HDAC6 (Abcam), and goat anti-rabbit IgG H&L (Alexa Fluor 488, Santa Cruz) secondary antibodies were employed.

## *In vivo* Model

### Animals

6-week-old female BALB/c mice (*n* = 5 in each experimental condition) were maintained for 1 weeks before starting the experiment. These mice were all housed under specific pathogen free (SPF) conditions. All animal experiments with mice were performed in accordance with the regulations and the approval of the Institutional Animal Care and Use Committee (IACUC) of Chung-Ang University (IRB Approval No. 2018-00097).

### Oxazolone-Induced Atopic Dermatitis-Like Skin in Mouse Ear

Hair of dorsal skin was shaved in all mice 2 days before starting sensitization. Following this, Mice were sensitized with 20 μl of 1% oxazolone or were applied with vehicle to the shaved dorsal skin. Four days after this sensitization, the ears of mice were repeatedly challenged with 20 μl of 0.1% oxazolone or vehicle, once every other day for 18 days. At the same time, after five times treatments of oxazolone, the mice were exposed to 100 μg/cm^3^ PM for 1 h a day in a closed-system chamber attached to a nebulizer (Macjin Medical, Republic of Korea). Ear thickness was measured with a digital caliper (Mitutoyo, Japan) before every challenge and immediately before euthanasia.

On sacrifice, ear skin and blood samples were collected after mice were anaesthetized using Avertin (Sigma-Aldrich). Oxazolone treatment and PM exposure condition were performed by modifications of previously described protocols [9].

### Statistical Analysis

The effects of PM and IL-4/13 on treatment groups and controls were compared using one-way analysis of variance (ANOVA) followed by Tukey's multiple-comparison post hoc test. Differences between groups were considered significant at *P* < 0.05. Statistical analyses were conducted using GraphPad Prism 8.0 (GraphPad Software, Inc., USA).

## Results

### Influence of PM on HDACs and FLG in AD-Induced HEKn Cells

**Fig. 1:**
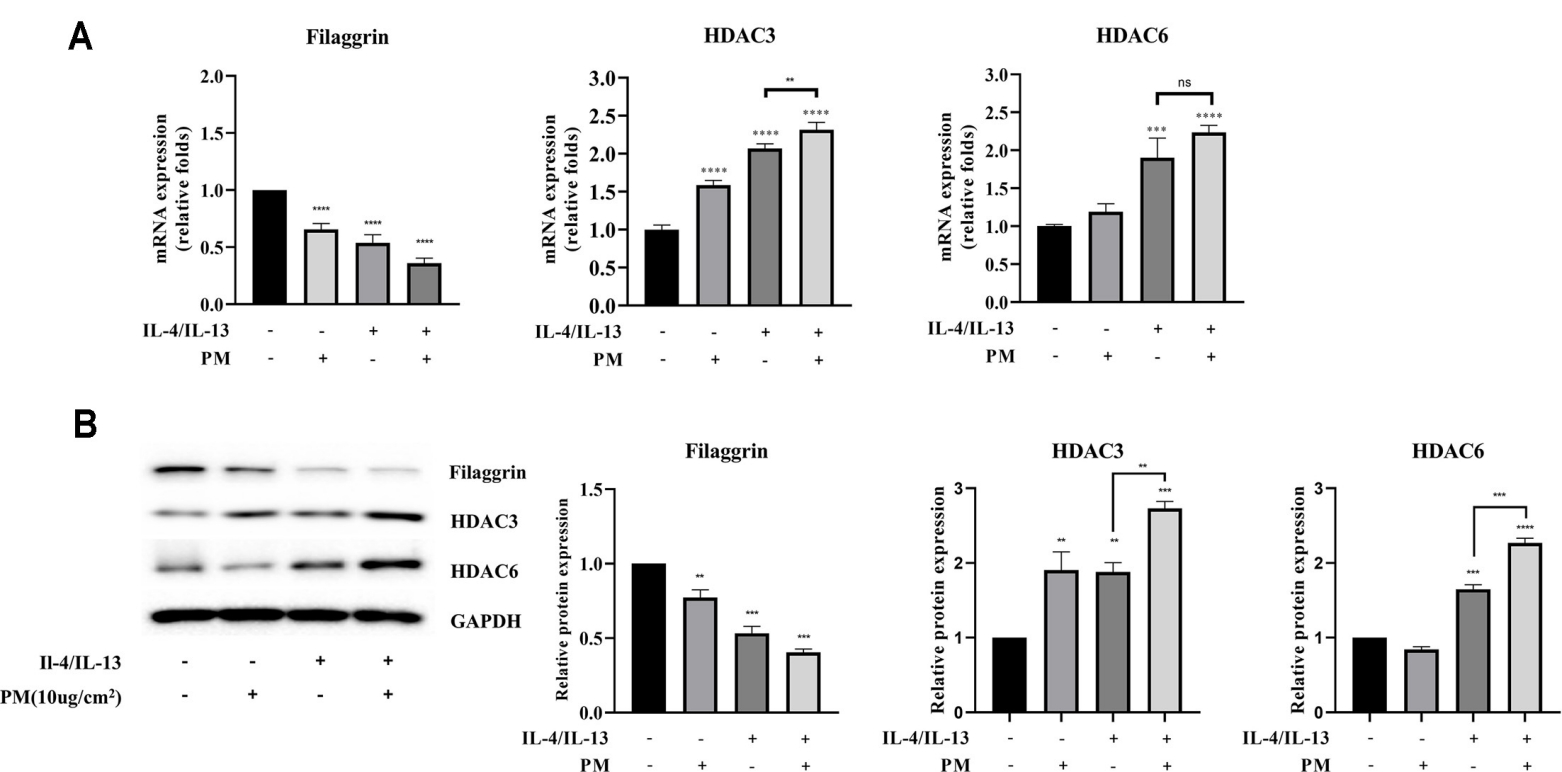
Effects of PM on HDAC and FLG in AD-induced HEKn cells. In the HEKn cell model, AD was induced with IL-4 (50 ng/ml) and IL-13 (50 ng/ml) and treated with PM (10 μg/cm^2^) for 24 h. (**A**) The HDAC3, HDAC6 and FLG mRNA expression levels were confirmed by qRT-PCR. (**B**) The protein expression levels of FLG, HDAC3 and HDAC6 were analyzed by western blot. The protein quantification results are illustrated. Data are presented as the mean ± standard deviation (*n* = 3) ****P < 0.0001, ***P < 0.001, **P < 0.01, *P < 0.05, compared with IL-4 + IL-13 levels.

At the mRNA level, FLG expression was decreased following treatment with either PM or IL-4/13 alone, with a further reduction upon combined treatment. HDAC3 and HDAC6 expression increased in response to IL-4/13 and PM, respectively, and showed further elevation with co-treatment (Fig. 1A). At the protein level, HDACs and FLG showed an opposite pattern (Fig. 1B).

Collectively, co-treatment with PM in AD-induced HEKn cells resulted in increase in HDAC3 and HDAC6. FLG displayed the most significant reduction when co-treated with PM in AD-induced HEKn cells.

### Effects of HDAC Inhibitors on HDACs and FLG in AD-Induced HEKn Cells with PM

To determine conditions for the HDAC inhibitor TSA, we treated HEKn cells with varying concentrations. FLG expression decreased in HEKn cells treated with TSA (Fig. S1).

**Fig. 2:**
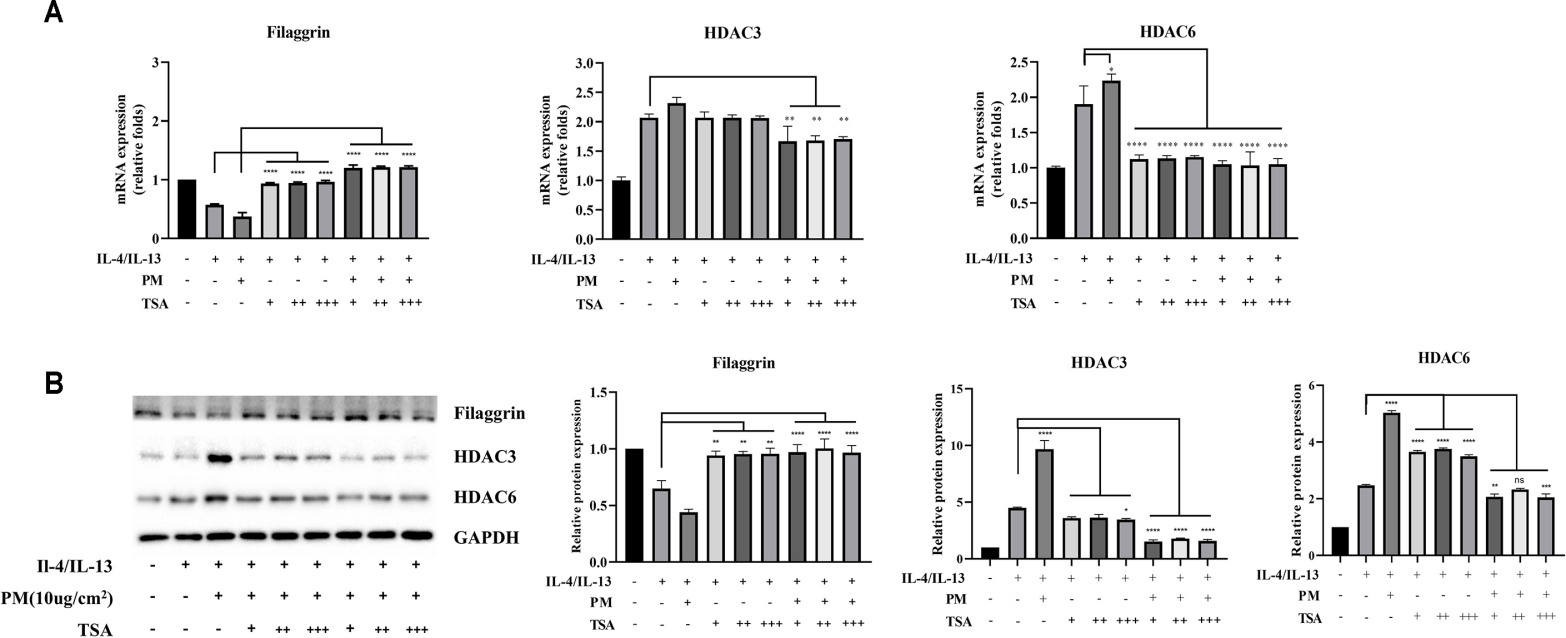
Effects of HDAC inhibitors on HDAC markers and FLG in AD-induced HEKn cells with PM. The HEKn cell model, in which AD was induced by IL-4 (50 ng/ml) and IL-13 (50 ng/ml), was pretreated with TSA (100, 200, and 300 nM) for 3 hours. Then, PM (10 μg/cm^2^) was treated for 24 h. (**A**) The HDAC3, HDAC6 and FLG mRNA expression levels were confirmed by qRT-PCR. (**B**) The protein expression levels of FLG, HDAC3, and HDAC6 were analyzed by western blot. The protein quantification results are illustrated. Data are presented as the mean ± standard deviation (*n* = 3). TSA (+: 100 nM, ++: 200 nM, +++: 300 nM). ****P < 0.0001, ***P < 0.001, **P < 0.01, *P < 0.05, compared with IL-4 + IL-13 levels.

**Fig. 3:**
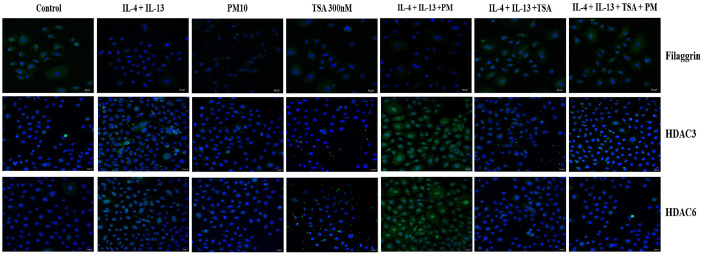
Immunofluorescence staining of HDACs and FLG in AD-induced HEKn cells with PM. The HEKn cell model, in which AD was induced by IL-4 (50 ng/ml) and IL-13 (50 ng/ml), was pretreated with TSA (300 nM) for 3 h. Then, PM (10 μg/cm^2^) was treated for 24 h. Green fluorescence is indicated by confocal images of immunofluorescent HDAC3, HDAC6 and FLG using FITC-conjugated rabbit anti-goat IgG H&L. The scale bar represents 20 μm.

FLG mRNA levels exhibited significant restoration when treated with TSA than with IL-4/13 or IL-4/13+PM co-treatment. HDAC3 and HDAC6 mRNA levels were much lower when TSA is given than when IL-4/13 and PM were given (Fig. 2A). The protein levels of HDAC3, HDAC6 and FLG were consistent with the mRNA expression profiles (Fig. 2B). Immunofluorescence analysis confirmed these findings (Fig. 3).

Overall, under AD-mimicking conditions with PM exposure, TSA effectively suppressed HDAC expression and restored FLG levels, indicating that HDAC inhibition can mitigate PM-induced epigenetic dysregulation in AD.

### Effects of PM on HDACs and FLG in AD-Induced *in vivo* Models

**Fig. 4:**
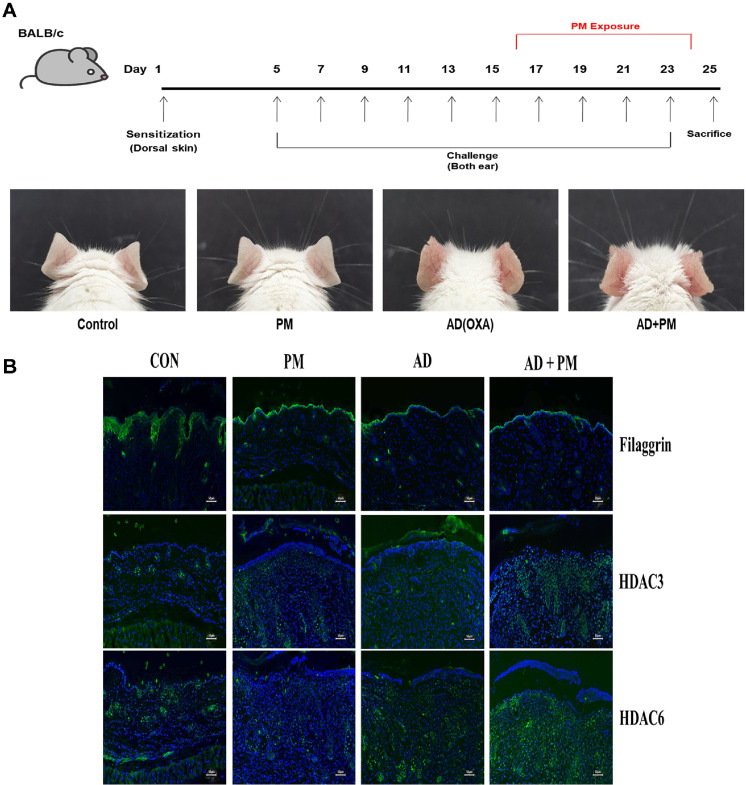
Immunofluorescence staining of HDAC2, HDAC3, HDAC6 and FLG in an oxazolone-induced dermatitis-like mice model. (**A**) Experimental protocol for atopic dermatitis. Clinical features were observed by photo images taken during the repeated application of oxazolone and SRM 1649b in mice model. (**B**) Immunofluorescence staining of control (normal mouse skin), PM, AD, AD+PM (scale bars 50 μm). Selected antibodies against histone deacetylation markers (HDAC3, HDAC6) and skin barrier marker (FLG).

To evaluate the in vivo relevance, immunofluorescence staining was performed to assess changes in FLG and HDAC expression in an AD-like mouse model exposed to PM (Fig. 4A). FLG expression showed a decreasing trend under PM exposure and AD conditions, with the most pronounced reduction observed under combined treatment. Conversely, HDAC3 and HDAC6 levels increased in the AD model, with a further rise upon PM co-exposure (Fig. 4B). These findings demonstrate that both PM and AD conditions modulate FLG and HDAC expression *in vivo*.

## Discussion

This study was conducted under the hypothesis that air pollutants, particularly PM, contribute to AD pathogenesis by inducing epigenetic alterations. This study aimed to determine whether PM suppresses FLG expression through HDAC-mediated regulation, thereby exacerbating AD phenotypes. Our findings showed that TSA treatment effectively restored FLG expression, supporting that PM exerts an epigenetic effect on FLG in AD [13, 25]. The highest expression of HDAC3 and HDAC6 was observed under co-treatment with IL-4/13 and PM and both were significantly reduced following TSA administration. These results suggest that HDAC3 and HDAC6 mediate PM-induced effects in AD-induced HEKn cells [21, 22].

The *FLG* expression was most suppressed under combined IL-4/13 and PM exposure and was substantially restored by TSA reinforcing the hypothesis that environmental pollutants disrupt skin barrier function through epigenetic mechanisms [13, 14].

HDACs are increasingly recognized as key regulators of gene expression in inflammatory and barrier-related skin disorders. Although earlier studies have suggested a role for HDAC6 in modulating STAT3 acetylation in allergic dermatitis [22], the direct relationship between HDACs and *FLG* expression has remained unclear. Our findings provide mechanistic evidence that HDAC3 and HDAC6 contribute to *FLG* suppression under Th2 cytokine stimulation and environmental stress, positioning HDACs as molecular integrators of immune and environmental signals in AD pathogenesis [3, 21].

The pan-HDAC inhibitor TSA, restored FLG expression while downregulating HDAC3 and HDAC6 in models exposed to PM and Th2 cytokines [25]. This finding indicates that HDAC activation is not merely a byproduct of inflammation but a functionally significant mechanism driving *FLG* suppression. Although TSA lacks isoform specificity, our findings align with previous reports highlighting its anti-inflammatory and barrier-restorative effects in allergic skin conditions [25].

The observed synergistic effect of PM and IL-4/13 in suppressing *FLG* and inducing HDACs is particularly significant, implying that the skin already compromised by inflammation is more vulnerable to environmental pollutants. This finding emphasized that patients with Th2-dominant immune responses are more susceptible to PM-induced barrier dysfunction, underscoring the need for environmental exposure management in AD treatment strategies [7].

Despite the valuable insights gained, this study has certain limitations. First, epigenetic mechanisms were inferred from gene and protein expression data. To determine whether HDACs directly regulate the *FLG* promoter, additional studies, such as chromatin immunoprecipitation (ChIP), histone acetylation assays, and transcriptomic profiling are warranted [15]. Second, although we used a triple-cell co-culture model incorporating keratinocytes, fibroblasts and mast cells to mimic the skin microenvironment, it does not fully replicate the complexity of human skin, such as the presence of Langerhans cells, dermal dendritic cells and the extracellular matrix. Third, our particulate matter exposure model utilized NIST SRM 1649b urban dust as a standardized particle source, which may differ in composition and physicochemical properties from real-world airborne particulate matter across diverse environments. Fourth, our *in vivo* experiments were performed in a murine model. Due to known anatomical and immunological differences between mouse and human skin, the translational relevance of these findings to clinical atopic dermatitis should be interpreted with caution. Future studies using human skin equivalents or clinical patient samples are warranted to further validate and expand these findings.

In addition, although TSA effectively reversed HDAC3/6-induced FLG suppression and restored skin barrier integrity in our model, its clinical application may be limited due to potential systemic side effects. TSA was originally developed as a broad-spectrum HDAC inhibitor for anti-cancer therapy and has been associated with adverse effects such as gastrointestinal disturbances, hematological abnormalities and immunosuppression in animal models and early-phase clinical trials [30, 31]. These off-target effects pose a practical limitation for long-term or systemic use in chronic skin diseases such as AD.

To overcome these limitations, developing TSA in a topical formulation or utilizing HDAC6-selective inhibitors (*e.g.*, Tubastatin A) may provide safer and more targeted therapeutic strategies [30, 32]. Moreover, advancements in nanocarrier-based drug delivery systems may enable localized delivery of TSA to the skin, thereby minimizing systemic absorption and improving treatment precision [32].

In conclusion, our findings demonstrate that PM impairs skin barrier function in AD through HDAC3 and HDAC6-mediated suppression of *FLG*, and that this effect can be reversed by the pan-HDAC inhibitor TSA [21, 22, 25]. However, further mechanistic studies are needed to elucidate how HDAC expression alters *FLG* regulation. As more epigenetic regulators implicated in AD pathogenesis are identified [3] new gene-regulatory therapeutic approaches are likely to emerge.

## Supplemental Materials

Supplementary data for this paper are available on-line only at http://jmb.or.kr.
