# Supplementary material for: Effect of Particulate Matter in Atopic Dermatitis through HDACs and Filaggrin Alteration
Source: J Microbiol Biotechnol. 2025 Jul 18;35:e2502047. doi: 10.4014/jmb.2502.02047 (PMC12324990; doi:10.4014/jmb.2502.02047)
Supplement: Supplementary file 1 [file jmb-35-e2502047-supple.pdf]

J. Microbiol. Biotechnol. 2025. 35: e2502047

https://doi.org/10.4014/jmb.2502.02047

Supplementary Figure

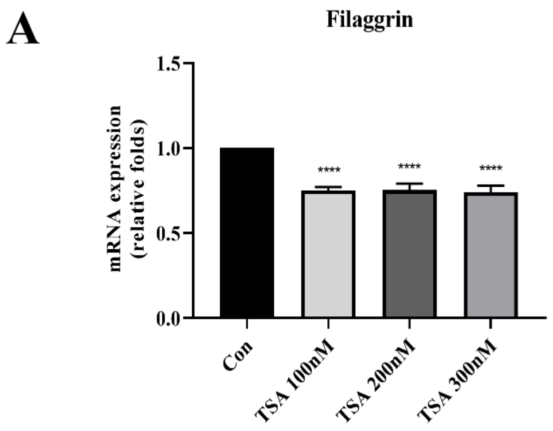

Fig. S1. Decreased FLG expression in HEKn cells treated with TSA. HEKn cells were pretreated with trichostatin A (TSA; 100, 200, 300 nM) for 3 hours, followed by FLG expression analysis by qRT-PCR. FLG expression levels were dose-dependently decreased upon TSA treatment. Data are presented as mean ± SD of triplicate experiments.
